# Optimizing Cancer Treatment: Exploring the Role of AI in Radioimmunotherapy

**DOI:** 10.3390/diagnostics15030397

**Published:** 2025-02-06

**Authors:** Hossein Azadinejad, Mohammad Farhadi Rad, Ahmad Shariftabrizi, Arman Rahmim, Hamid Abdollahi

**Affiliations:** 1 Department of Immunology, School of Medicine, Kermanshah University of Medical Sciences, Kermanshah 6714869914, Iran; azadinejad.hossein@gmail.com; 2 Radiology and Nuclear Medicine Department, School of Paramedical Sciences, Kermanshah University of Medical Sciences, Kermanshah 6715847141, Iran; 3 Department of Radiology, University of Iowa Hospitals and Clinics, Iowa City, IA 52242, USA; ahmad-shariftabrizi@uiowa.edu; 4 Department of Radiology, University of British Columbia, Vancouver, BC V6T 1Z4, Canada; 5 Department of Integrative Oncology, BC Cancer Research Institute, Vancouver, BC V5Z 0B4, Canada; 6 Department of Physics and Astronomy, University of British Columbia, Vancouver, BC V6T 1Z4, Canada

**Keywords:** radioimmunotherapy, cancer treatment, personalized medicine, monoclonal antibodies

## Abstract

Radioimmunotherapy (RIT) is a novel cancer treatment that combines radiotherapy and immunotherapy to precisely target tumor antigens using monoclonal antibodies conjugated with radioactive isotopes. This approach offers personalized, systemic, and durable treatment, making it effective in cancers resistant to conventional therapies. Advances in artificial intelligence (AI) present opportunities to enhance RIT by improving precision, efficiency, and personalization. AI plays a critical role in patient selection, treatment planning, dosimetry, and response assessment, while also contributing to drug design and tumor classification. This review explores the integration of AI into RIT, emphasizing its potential to optimize the entire treatment process and advance personalized cancer care.

## 1. Introduction

Cancer remains a leading cause of death globally, with over 19 million new cases and 10 million deaths annually, accounting for one-sixth of all deaths worldwide. By 2040, cancer incidence is projected to exceed 28 million cases, with a more significant rise in transitioning countries [1,2]. Traditional treatments like surgery, chemotherapy, and radiotherapy have achieved progress but often cause collateral damage to healthy tissues [3,4]. Radiopharmaceutical therapy (RPT) has emerged as a groundbreaking solution, targeting cancer cells with radioactive substances while sparing healthy tissues [5]. This precise, targeted approach is particularly effective in advanced or resistant cancers, such as prostate, thyroid, and neuroendocrine tumors, improving symptoms, quality of life, and survival rates [6]. With ongoing advancements, RPT is poised to play a critical role in the future of cancer care [7].

Radioimmunotherapy (RIT), introduced in the 1950s, is a form of radiopharmaceutical therapy (RPT) that uses immune proteins as radioisotope transporters, tracers, or targeted therapeutics. Administered intravenously or compartmentally, RIT employs radionuclides such as alpha, beta, and auger electron emitters. While intact monoclonal antibodies are commonly used, newer fragments like Fab, nanobodies, and affibodies have shown potential to enhance efficacy [8,9,10].

RIT has been particularly successful in treating hematological cancers due to the availability of cell surface antigens, advanced antibody development, and the radiosensitivity of leukemia and lymphoma. Clinical trials with 90Y-ibritumomab demonstrate progression-free survival rates of 26–40.2 months [11,12]. However, its efficacy in solid tumors is limited by poor vascularization, restricted diffusion, and lower emitted radiation doses to target sites [12,13].

**Figure 1:**
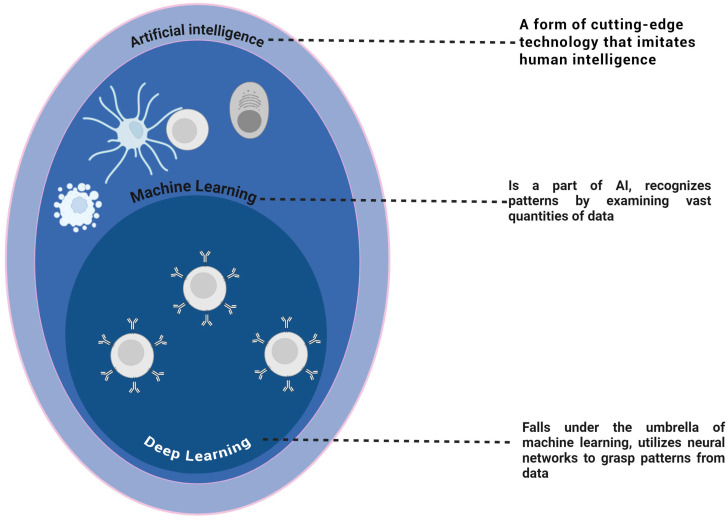
Visual overview of artificial intelligence (AI) and its subdisciplines, machine learning (ML) and deep learning (DL). Both ML and DL are subsets of AI. ML uses vast amounts of data to recognize patterns, while DL uses neural networks to group data based on particular patterns.

Artificial intelligence (AI) holds transformative potential to revolutionize radioimmunotherapy (RIT) by employing advanced algorithms to analyze complex datasets, identify patterns, and optimize the treatment process. Machine learning (ML), a fundamental branch of AI, and its subset, deep learning (DL), enable sophisticated tasks such as tumor detection, treatment planning, and drug discovery. DL techniques, including convolutional and recurrent neural networks, excel at processing high-dimensional data, driving significant advancements in cancer research [14,15,16,17,18,19,20,21,22]. Figure 1 illustrates the hierarchical relationship between AI, ML, and DL, emphasizing their roles in analyzing data and extracting complex patterns to advance personalized cancer care.

In cancer care, AI improves detection, diagnosis, and treatment by enabling rapid analysis of big data and supporting precision medicine strategies that tailor therapies to individual patients [23,24,25,26,27]. This review explores the integration of AI into RIT, highlighting its potential to optimize the process and advance personalized cancer treatment.

This narrative review includes a detailed methodology section to enhance transparency and credibility. We conducted an extensive literature search across major databases such as PubMed, Google Scholar, and Scopus, and Web of Science to ensure comprehensive coverage of relevant advancements. The search utilized specific keywords like “radioimmunotherapy,” “artificial intelligence,” “radiopharmaceuticals,” and “personalized cancer treatment,” with Boolean operators to refine and combine terms effectively. Filters were applied to focus on peer-reviewed articles published in English over the past 20 years, with a few seminal studies from earlier years (e.g., 2003 and 2004) included for their continued relevance. This rigorous process initially identified about 300 papers. After a thorough evaluation of their relevance and quality, approximately 200 impactful studies were selected as the foundation for this review, ensuring a robust synthesis of both current and foundational knowledge.

## 2. RIT Process

**Figure 2:**
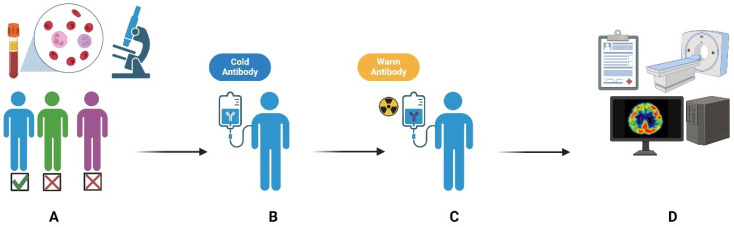
Radioimmunotherapy process at a glance. (**A**) Selection of eligible patients based on history and lab tests. (**B**) Administration of non-radioactive mAb via IV for tissue protection. (**C**) Injection of radiolabeled mAb as the main treatment in the following week. (**D**) Conclusion with imaging and lab tests to assess tumor response and treatment efficacy (further elaborated in the text).

RIT is a targeted cancer treatment that combines the specificity of monoclonal antibodies (mAbs) with the cytotoxic effects of radiation. It utilizes radiolabeled antibodies that can selectively bind to specific cancer cell antigens, delivering localized radiation directly to cancer cells while sparing healthy tissues [28]. This innovative approach has shown promising results in the treatment of various hematological malignancies and solid tumors, offering a potential avenue for personalized and targeted therapy in oncology [4]. Figure 2 illustrates the RIT steps. Recently, RIT has gained popularity because it utilizes small molecules like peptides instead of antibodies, allowing for faster and easier penetration into tissues [29].

Patient selection is crucial for RIT. Candidates should be free of certain allergies, avoid specific medications, and have normal hematological parameters. Those with recent bone marrow transplants, pregnant women, and children are not suitable for RIT [30]. RIT is typically an outpatient procedure with multiple hospital visits. The first visit involves receiving a dose of the monoclonal antibody and the radiotracer injection, with subsequent visits for imaging and treatment scheduling [31]. The actual treatment, given seven to nine days after the initial scan, consists of an IV infusion of radioimmunoconjugates [32].

## 3. Radionuclides

Approximately 20 radionuclides among 1200 isotopes of elements have optimal characteristics (chemical, physical, and pharmacological, including physical half-life (t1/2), availability, decay energy, etc.) to be considered as RIT radionuclides with monoclonal antibodies [33]. Three types of radionuclides can be used in RIT, emitting α-particles, β-emitters, and/or auger electrons. These radionuclides have some advantages and limitations regarding their physical and radiobiological properties (Table 1). Mean energy, half-life, range in tissue, and linear energy transfer (LET) are vital properties that influence the damage produced by radionuclides [34].

**Figure 3:**
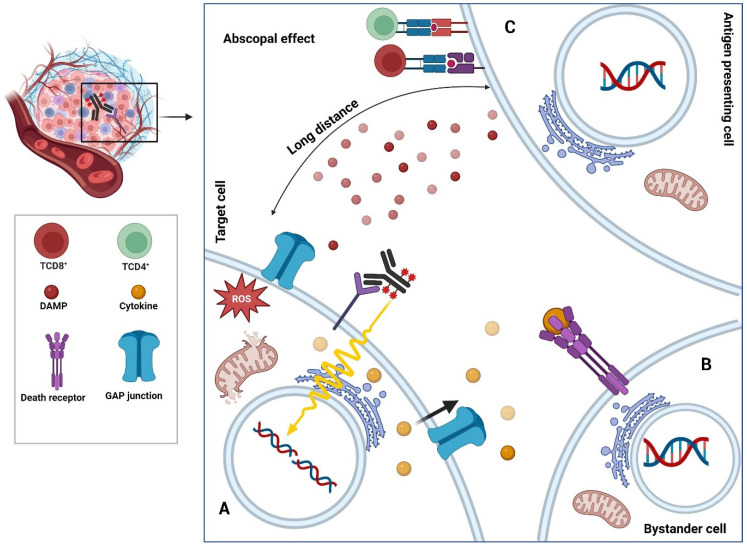
Cell mechanisms underlying radioimmunotherapy. (**A**) The crossfire effect results in the exposure of the adjacent cells to radiation. (**B**) Bystander effects occur due to the interactions between the targeted and adjacent cells. (**C**) The abscopal effect is characterized by the demise of cells distant from the targeted cell that are not the primary focus of treatment.

Along with the inherent characteristics of radionuclides, RIT also produces a variety of therapeutic outcomes based on the features of the used antibody. As shown in Figure 3, radionuclides have three effects: (1) crossfire radiation, which causes juxtracrine cells to be exposed to radiation (Figure 3A); (2) bystander effects, which happen as a result of interactions between the targeted and nearby cells (Figure 3B); and (3) the abscopal effect, namely the death of cells that are not targeted but are located far from the targeted cells (Figure 3C) [10].

α-particle and Auger electron-emitting radionuclides are desirable for RIT of cancers due to their potential for increased efficacy and lower non-specific toxicity to non-targeted normal tissues [43,44]. Matching the radioisotope physical half-life with the pharmacophore biological half-life increases the contrast ratio for imaging or irradiation dose to lesions for therapy. Based on half-life time, different radionuclides are appropriate for other mAbs. Radioisotope selection is further influenced by variables such as radioisotope availability, cost, and production process [45].

## 4. Applications of RIT

Radioimmunotherapy (RIT) has established its significance in managing hematological malignancies, particularly non-Hodgkin’s lymphoma (NHL). The FDA-approved radioimmunoconjugate 90Y-ibritumomab tiuxetan (Zevalin), administered intravenously, effectively delivers systemic radiation to target and bystander CD20-expressing cells and has been used successfully for relapsed or persistent cancers [46]. Clinical studies demonstrate that the complete response rates for 90Y-ibritumomab tiuxetan range from 15% to 38%, with overall response rates between 74% and 83% [12]. However, significant adverse effects, including myelosuppression, have been observed, affecting 95% of patients with anemia, 77% with neutropenia, and 61% with thrombocytopenia, though most side effects are transient, necessitating supportive care for some [12,47].

Another pertinent agent is tositumomab, a murine monoclonal antibody against CD20, combined with iodine-131 to create the radiopharmaceutical Bexxar. Approved in 2003 for relapsed or chemotherapy-resistant NHL, Bexxar is associated with severe, prolonged reductions in blood cell counts and related complications, including infections and bleeding, as well as allergic reactions, secondary leukemia, and myelodysplasia [48,49,50]. Despite the potential of these treatments, the hematology-oncology community’s limited embrace has led to the commercial decline of both Zevalin and Bexxar, culminating in the discontinuation of Bexxar production in the U.S. in 2014 [51]. Research continues beyond B-cell lymphoma, targeting other hematological malignancies with promising developments utilizing markers like CD33, CD45, and CD66, in addition to alpha emitters such as Bi-212 and Ac-225, which show better efficacy than beta emitters in cancers like acute myeloid leukemia (AML) [52].

In contrast, the therapeutic index of RIT in solid tumors remains inadequate due to the inflammatory, fibrotic nature of most solid tumors that create biophysical and metabolic stress, impairing vascular transport and resulting in heterogeneous radio-conjugate delivery [53,54]. The radiation burden from RIT frequently limits the dose to the bone marrow. To enhance RIT effectiveness, strategies such as compartmental administration have been explored; injecting radioimmunoconjugates into compartments like the intrathecal space can yield up to ten-fold more radiation delivery to tumors compared to systemic delivery [4,12]. Moreover, efforts to improve RIT for solid tumors include investigations by Meredith et al. in 2001, which demonstrated that combining the radiolabeled mAb [177Lu] Lu-CC49 with chemotherapy was well-tolerated, with bone marrow suppression acting as the dose-limiting factor [55]. Additionally, pretargeted radioimmunotherapy (PRIT) is under investigation, showing promise in enhancing the therapeutic index for solid tumors based on preclinical results [9].

## 5. Pretargeted RIT (PRIT)

**Figure 4:**
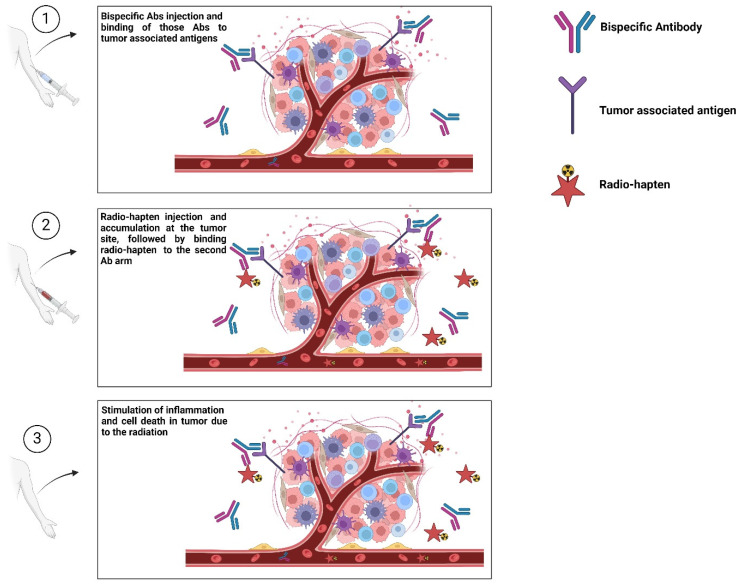
Pretargeted radioimmunotherapy process. (**1**) A bispecific antibody is injected into the patient, binding to a tumor-associated antigen with one arm. (**2**) During a subsequent visit, after the accumulation of the bispecific antibody at the tumor site, a radiolabeled hapten is administered to the patient, binding to the antibody’s other arm. (**3**) Radiation from the radiolabeled hapten leads to inflammation and lysis of tumor cells.

As mentioned earlier, despite the success of radioimmunotherapy in treating hematological tumors, conventional radioimmunotherapy has been less successful in treating solid tumors. Fundamentally, the dose-limiting toxicities, poor mAb uptake, and antidrug antibodies prevented radioimmunotherapy of solid tumor masses from becoming successful [4]. PRIT significantly enhances the Tis (tumor-to-normal tissue absorbed dose ratios) by separating the tumor targeting and radio carrier (e.g., a radiohapten) administration phases. It is possible to engineer the tumor-targeting bispecific protein to enhance tumor uptake and therapeutic index (TI). Creating a chase molecule or clearing agent (CA) to transport unbound proteins from the blood to the liver for degradation is possible. Following an ideal pretargeting interval of hours or days, an intravenous cargo with high affinity for the second specificity in the protein explores the bispecific protein connected to the tumor or clears from the body in minutes to hours [56]. In addition to the antibodies with dual specificity, PRIT has three more advanced generations. In the following, we will briefly explain the three new generations of PRIT and the recent developments in this field. Figure 4 shows the PRIT process.

### 5.1. Biotin-Streptavidin Approaches and the Potential of PRIT

Although PRIT with BsAb showed great potential, inappropriate TIs caused by low affinity or avidity for the hapten led to the discovery of alternatives. Streptavidin and avidin are tetrameric proteins, with each subunit capable of binding a single biotin molecule with comparable affinities. The biotin-streptavidin system was an appealing possibility for pretargeting because of its much higher affinities [56].

Recently in 2015, a trial with B9E9FP (anti-CD20-streptavidin fusion protein that has been genetically created) and 111In/90Y-biotin was launched in patients with high-risk B-cell malignancies to assess the safety of combining PRIT with carmustine, etoposide, cytarabine, and melphalan chemotherapy, as well as autologous stem cell transplantation (NCT02483000).

### 5.2. Second-Generation BsAb PRIT with Multivalent Haptens

Along with the introduction of biotin-streptavidin PRIT in the late 1980s, researchers sought to improve contrast (for pretargeted radioimmunodiagnosis) by enhancing hapten selectivity for intratumoral BsAb over circulating BsAb [56]. A clinical trial aimed to refine the dosing of reagents and the timing of administration for humanized bispecific antibodies (BsAb) in 35 patients suffering from CEA-expressing tumors. The doses of BsAb varied between 10 and 100 mg/m^2^, while the doses of 131I-diDTPA(indium)-hapten ranged from 1.9 to 5.5 GBq. Additionally, pretargeting intervals of either 5 or 7 days were evaluated. With the optimized radioimmunotherapy (PRIT), specifically using 40 mg/m^2^ of BsAb and a 5-day pretargeting interval, doses of 131I-diDTPA(indium)-hapten up to 5.5 GBq were shown to be well tolerated when there was no bone marrow involvement [57]. In a follow-up study involving 22 patients with CEA-expressing tumors, researchers varied the doses of humanized bispecific antibodies (BsAb) (ranging from 40 to 75 mg/m^2^) and the doses of 131I-diDTPA(indium)-hapten (from 1.8 to 2.9 GBq/m^2^; 1.9 to 5.5 GBq) to assess their antitumor effectiveness and associated toxicity. Myelosuppression was found to be dependent on the BsAb dose; the 75 mg/m^2^ dose caused significant hematologic toxicity, while non-hematologic toxicities were primarily hepatic and transient (grade I or II) [58]. At the 75 mg/m^2^ BsAb dosage, the average radiation doses to the whole body and liver were higher (0.38 Gy and 1.9 Gy, respectively) compared to the 40 mg/m^2^ dose (0.33 Gy for the whole body and 1.4 Gy for the liver). However, there was no significant difference in mean tumor doses between the doses, with 75 mg/m^2^ resulting in an average of 10.7 Gy (range 1.7–53.5 Gy) and 40 mg/m^2^ yielding 18.5 Gy (range 2.4–49.3 Gy). The reported therapeutic efficacy was modest, with no complete or partial responses observed [58]. The maximum tolerated dose (MTD) for the 131I-diDTPA(indium)-hapten in patients with medullary thyroid carcinoma (MTC) was established at 3 GBq, while it was not defined for non-MTC patients, who were escalated beyond 5.5 GBq. Additionally, an elevation of human antimouse antibodies was noted in one patient (8%), and human antihuman antibodies were found in four patients (33%) [56,58].

### 5.3. Third-Generation BsAb PRIT with Anti–Histidine-Succinyl-Glycine (HSG) mAb and BsAb Prepared via Dock and Lock (DNL)

Because of mAb specificity, the anti-111In-DTPA hapten method could not be employed to target 90Y or 177Lu [59]. Despite the fact that more anti-chelate mAbs were produced, anti-2,4-dinitrophenyl and anti-HSG pseudo peptide mAbs were tested as potential anti-hapten mAbs for PRIT [60]. Sharkey et al. enhanced the HSG system for PRIT significantly in 2003 by inventing a novel BsAb (e.g., anti-CEA F(ab’)_2_ or anticolon-specific antigen-p F(ab’)_2_ chemically coupled to antiHSG Fab) and a suite of HSG peptides for targeting a number of clinically relevant radionuclides (e.g., IMP241 for 90Y, 111In, and 177Lu and IMP245 for 99mTc and 188Re) [61].

The DNL system has been widely researched in preclinical (mainly for creating new haptens) and clinical settings, with many trials involving DNL anti-CEA TF2 and radiolabeled IMP288. RDC018 is a novel radiolabeled/near-infrared multimodal DNL hapten for image-guided surgery of different carcinomas, while 213Bi-IMP288 is an α-PRIT hapten [62,63].

### 5.4. Making Use of Affibody Molecules as PRIT Vectors

Although IgG-based mAbs and BsAbs have typically been utilized as PRIT vectors, their extended circulation can make complete clearance difficult to achieve, potentially resulting in unintended bystander toxicity. Protein engineering advances have resulted in novel protein constructions such as mini bodies (80 kDa), diabodies (50 kDa), and designed scaffold proteins (4–20 kDa). These tiny structures are beneficial as radionuclide imaging vectors [64]. However, Affibody molecules have been proven to be suboptimal as RIT vectors for residualized radiometal labels, resulting in significant renal absorption [65].

### 5.5. Pretageting Using Biorthogonal

Any chemical reaction that can happen inside biological systems without interacting with native biochemical processes is called biorthogonal [66]. PRIT has also successfully employed trans-cyclooctene-modified mAbs and radiolabeled tetrazine for inverse electron-demand Diels–Alder. PRIT was demonstrated in mice with TAG-72-expressing human colorectal cancer xenografts with ^212^Pb-tetrazine in 2017 [67].

## 6. Biomarkers for RIT

Three types of immune biomarkers can be used in RIT: tumor microenvironment, genomic, and peripheral blood levels. These biomarkers can be utilized to improve RIT efficacy, predict tumor prognosis, evaluate tumor response to irradiation, and select patients [68].

### 6.1. Tumor Microenvironment Level

Numerous modifications can be brought about in the tumor microenvironment by ionizing radiation. When radiation is administered to the tumor bed, healthy stromal cells and cancer cells may undergo phenotypic changes that alter their physiological and molecular characteristics. The tumor microenvironment immunogenicity level is directly influenced by these environmental modifications. For example, tumor vasculature and intratumoral perfusion can be affected by radiation. The microvascular network plays a crucial role in regulating tumor growth and has emerged as a promising target for cancer therapy. Radiation directed at tumors can trigger responses in endothelial cells, which can influence both its effectiveness against tumors and its harmful effects on healthy tissue. In addition, radiation impacts the immunogenicity of tumor cells by triggering immunogenic cell death (ICD) and also influences the tumor microenvironment by stimulating the release of various cytokines in the tumor tissue. These cytokines include epidermal growth factor, proinflammatory cytokines, fibroblast growth factor, and transforming growth factor (TGF) [69].

### 6.2. Genomic Level

Recent studies have emphasized the potential connections between the tumor genome and the effectiveness of immunotherapy, showing that the genetic background would affect the immuno-editing procedure. Tumor mutational landscape and neoantigen load are two possible immune biomarkers for RIT at the genomic level. The capacity of IR to cause irreparable DNA lesions, mostly double-strand breaks within the tumor cell, is crucial to its radiobiological efficacy. As a result, a cell goes through several procedures to detect and repair damage to the DNA molecules that encode its genome. [70] Research has shown that IR can induce immunogenic cell death (ICD) by releasing danger-associated molecular patterns (DAMPs), which are identified by the innate immune system. These DAMPs play a crucial role in enhancing the presentation of tumor-associated antigens to T lymphocytes via the major histocompatibility complex (MHC) of antigen-presenting cells (APCs) [68].

### 6.3. Peripheral Blood Immunomonitoring Level

Soluble immune-connected and cellular biomarkers are two possible immune biomarkers at the peripheral blood level for RIT. Peripheral biomarkers might be non-invasive, simple to repeat, and theoretically less expensive compared to tumor biopsy. Numerous cellular biomarkers should be evaluated in different cancers and patients to correlate the presence of immune biomarkers in the peripheral blood and the strategy we are working on [68].

## 7. Dosimetry

Nuclear medicine uses radionuclides for several diagnostic and treatments. To assess the dangers and advantages of any procedure, one must be aware of the radiation dose that various body organs receive [71].

Human internal dosimetry following the integration of radioactivity is a difficult task since it is impossible to measure the committed doses. Thus, activities retained in the body or in body excretions (urine and feces) should be measured at specific times after ingestion. Biokinetic and dosimetric models can be used to measure internal exposures. Biokinetic models detail the retention and excretion of radionuclides and their metabolic behavior in the body. Dosimetric models, however, describe the energy deposition that occurs when radionuclides and their progeny decay. Numerous sources of uncertainty are present during the entire dose assessment procedure. Structured methodologies for creating appropriate monitoring programs and evaluating the monitoring data have been established to guide the assessors and lessen the uncertainties brought on by various techniques and assumptions used in the calculations [72].

To investigate particle track structure in various target volumes and to determine whether it correlates with the biological consequences of various radiation quality, Monte Carlo (MC) simulation techniques are frequently used [73]. Critical benefits of MC simulations include the capacity to consider an uneven distribution of radioactivity, the generation of secondary particles (typically γ-radiation), the transitions between different tissue types, and patient-specific organ and lesion geometries. Many studies use MC simulations to test novel, quicker algorithms for specific activity distribution, absorption, crossfire, and tissue transition assumptions [74,75].

In the late 1960s, the Medical Internal Radiation Dose (MIRD) Committee of the Society of Nuclear Medicine developed a formalism for computing the absorbed dose for medical applications of radiopharmaceuticals.

$${\bar{D}}_{rT}={\tilde{A}}_{rs}\;S_{rr\leftarrow rs}$$

where *A* is the cumulated activity, *D* is the mean absorbed dose to a target volume, and *S* is a variable that describes the mean absorbed dose to the target volume per unit cumulative activity in the source volume. For radionuclides administered internally, the cumulative activity is influenced by the radionuclide biological half-life (governed by the radionuclide’s biokinetic behavior) and the physical half-life of the radionuclide. In addition, MC simulations have been used to calculate *S* values for many isotopes. The source-to-target separation, tissue density, target mass, and radionuclide emission spectrum affect the S value [76,77]. Due to relatively quick and easy algorithms that only require successive 2D imaging to determine activity distributions and the use of average organ features, S value dosimetry is suitable for clinical usage. Despite the above presumptions, this method has become the industry-standard dosimetry method for pharmaceutical investigations. Tumor dosimetry is feasible; however, it has some limitations, e.g., tumor lesions are considered spherical and crossfire dosage is not considered [77].

Creating novel 3D dosimetry techniques for targeted radionuclide therapy has attracted much interest. The drawbacks of using pre-calculated energy transfer coefficients produced by virtual anthropomorphic phantoms sparked this interest. “Voxel-wise dosimetry” aims to estimate the dose or dose rate for every voxel contained in a nuclear medicine imaging volume. Convoluting an isotope-specific energy deposition kernel with the activity map is the simplest way to use dose point kernel voxel-wise dosimetry [78]. A voxel-by-voxel dose map is produced by calculating the mean absorbed dose for each voxel using a dose kernel matrix (mGy MBq 1 s 1). The capability of addressing heterogeneous radioactivity distributions at the organ or tumor level is a benefit of dose kernel dosimetry. Additionally, to evaluate radiobiological effects, 3D dose distributions enable the visualization of isodose lines and dose-volume histograms (DVHs) [79,80].

In local energy deposition, all energy is assumed to be absorbed in the voxel of origin. Due to the deeper penetration of γ-emissions and secondary photons, this idea does not apply. However, for some α- and β-particles or auger electrons, it is true. However, this method is pretty accurate for a rapid study, such as toxicity studies, if one is primarily interested in evaluating particular regions of the radionuclide emission spectrum [81,82,83].

## 8. Evaluation of Tumor Response in Radioimmunotherapy

Tumor response is the evaluation of the response to the treatment. As new methods for cancer treatment continue to enter routine oncology practice, tumor response assessment must evolve to capture their actual biological effects. In contrast to traditional cancer therapy imaging, where cross-sectional measurements often correlate with response, tumor edema and inflammation after immunotherapy may confound traditional approaches to response assessment. Whole-body MRI offers excellent diagnostic performance for assessing therapy response in hematological malignancies [84]. Emerging evidence suggests that circular RNA dysregulation is associated with carcinogenesis and therapy response in hematological malignancies [85]. In solid tumors, imaging assesses traditional treatments such as chemotherapy. Cross-sectional imaging, however, cannot be employed in immunotherapy because of the edema and inflammation induced in the tumor following this form of treatment. The possibility of swelling resulting from a successful immune response can lead to deceptive imaging findings, especially in the short term [84].

Clinicians and researchers recognized the need for standardized radiographic response criteria after introducing chemotherapy and radiation therapy for solid tumors [86]. Numerous groups created practical techniques for classifying solid tumor therapy responses based on tumor size and number changes to meet this need. These have been adjusted when new data and treatment regimens have become available, including attempts to address immunotherapy responses correctly [84].

The RECIST (Response Evaluation Criteria in Solid Tumor) recommendations were published in 2000 by a multi-national task team. The RECIST 1.0 recommendations describe particular target lesions assessed to establish total tumor burden. The number of target lesions was increased in RECIST 1.1, as were lymph node assessments and suggestions for validating each response category (response categories include a complete response, partial response, no change, and progressive disease) [87,88].

RECIST 1.1 was the foundation for iRECIST (immune RECIST). Target lesions can be up to five detectable tumors, with no more than two for each organ. To determine response, new lesions are evaluated separately from the original. iRESIST is very well adapted to immunotherapy. iRECIST also includes a comprehensive guideline for taking clinical symptoms into account. The criteria emphasize the importance of examining the patient’s state in addition to radiographic results before determining treatment decisions [84,89].

## 9. AI-Driven Approaches in Early Cancer Detection and Molecular Imaging

Artificial intelligence is increasingly facilitating the early identification of cancer using emerging minimally invasive methods like liquid biopsies that assess circulating tumor DNA (ctDNA) or cell-free DNA (cfDNA). This process, which involves straightforward blood tests, theoretically permits the early diagnosis of cancer, continuous monitoring of relapse risks, and the tailoring of treatment plans. For example, ctDNA analysis can predict microsatellite instability (MSI) in endometrial cancer patients, thus aiding in the selection of immunotherapy regimens [90]. Chabon et al. [91] introduced a machine learning approach known as Lung-CLiP that forecasts the probability of finding circulating tumor DNA (ctDNA) in blood collected from individuals with lung cancer. Chabon et al.’s method begins by determining the likelihood that a mutation in cell-free DNA (cfDNA) is tumor-associated, employing an elastic net model that includes characteristics such as cfDNA fragment size. It then merges this model’s output with copy number scores in an ensemble classifier that deploys five different algorithms to predict the presence of circulating tumor DNA (ctDNA) in blood samples. The method exhibited moderate predictive capabilities (AUC = 0.69–0.98), influenced by the stage of cancer and demonstrating a tradeoff between sensitivity and specificity. In a related study, Mouliere et al. [92] reported on a random forest classifier that utilizes features based on cfDNA fragment sizes to predict ctDNA presence with significant accuracy across multiple cancer types (AUC = 0.91–0.99). Cohen et al. [93] developed CancerSEEK, an end-to-end blood test that detects early-stage cancer and identifies one of eight specific cancer types from circulating tumor DNA (ctDNA). The process begins with a logistic regression model that determines whether a sample is cancer-positive by examining mutations in 16 genes and the levels of eight plasma proteins. Following this, a random forest classifier predicts the specific cancer type, achieving accuracy rates ranging from 39% to 84% based on the type of cancer. This research is particularly impactful since five of the eight cancer types included in the test currently have no early screening options available. Overall, while advancements in AI for early cancer detection are significant, they have mostly relied on conventional machine learning methods. With the expansion of data acquisition from liquid biopsies, more advanced deep learning frameworks are anticipated to minimize the reliance on manual selection and curation of key features. Furthermore, future use of multi-modal strategies, like CancerSEEK, which incorporate diverse data types such as liquid biopsies and imaging, will likely improve early detection and facilitate ongoing monitoring of disease risk over time [94].

PET is a powerful, non-invasive imaging modality with diverse applications in both clinical practice and research. It generates three-dimensional images of organs and lesions using radioactive tracers, which can be attached to substances that the organs typically utilize, such as glucose, or to molecules that bind to cellular receptors, peptides, or cytokines [95,96]. Recent innovations in PET technology and software have improved imaging speed and sensitivity [97]. The integration of the precise targeting capabilities of immune-related molecules with the PET technique’s inherent sensitivity has led to the emergence of ImmunoPET, which can specifically target various molecular pathways involved in tumor biology [98]. A wide range of tumor-targeting vectors have been studied for ImmunoPET, with full-length antibodies (Abs) ranking among the most frequently used [99].

Tumor cells and their surrounding environment exhibit increased levels of certain proteins that aid in tumor proliferation and invasion. These proteins are significant for identifying tumor types and are suitable targets for nuclear imaging in cancer diagnosis. An example includes radiopharmaceuticals that focus on prostate-specific membrane antigen, which is prominently expressed in prostate cancer and supporting blood vessels, allowing for precise staging of the disease [100]. Fibroblast activation protein, which is predominantly overexpressed in cancer-associated fibroblasts, is a promising pan-cancer target useful for diagnosing a wide range of tumors [101]. This line of research has gained significant traction in China. In addition to these well-recognized targets, research is underway on radiotracers that target novel biomarkers in preclinical and translational studies, including those that have been significantly advanced internationally, such as carbonic anhydrase IX [102], integrin αvβ6 [103,104], and CD38 [105,106].

AI is revolutionizing ImmunoPET. The integration of sophisticated machine learning algorithms with traditional imaging techniques significantly boosts the precision and efficiency of imaging processes. One major use of AI in ImmunoPET is to improve image reconstruction techniques. Conventional reconstruction methods, including filtered backprojection and iterative algorithms, have limitations in terms of resolution and noise management. AI-driven techniques apply deep learning and multilayer neural networks to refine these processes, leading to enhanced image quality through methods such as denoising, super-resolution refinement, and inferred attenuation correction. These enhancements facilitate more effective tumor detection and precise evaluation of treatment responses [107]. AI significantly enhances the field of radiomics by evaluating and quantifying connections within medical imaging data. By extracting features from PET images, AI can detect intricate patterns related to distinct disease processes or patient outcomes [108]. This functionality facilitates the formation of accurate imaging phenotypes, which are vital for tailoring treatment plans and predicting the likely effectiveness of certain therapies for individual patients.

While ImmunoPET offers significant advantages, such as high specificity in cancer diagnosis, it also faces challenges that can limit its effectiveness. Key issues include the validation and generalizability of AI models, as many studies rely on limited retrospective data from single institutions, hindering broader applicability. Additionally, there are concerns regarding data variability, insufficient methodological rigor, ethical and data security implications, and institutional barriers that impede multi-site collaboration, all of which can compromise the reliability and utility of ImmunoPET in diverse clinical settings [108,109,110,111].

## 10. AI in the RIT Process

**Figure 5:**
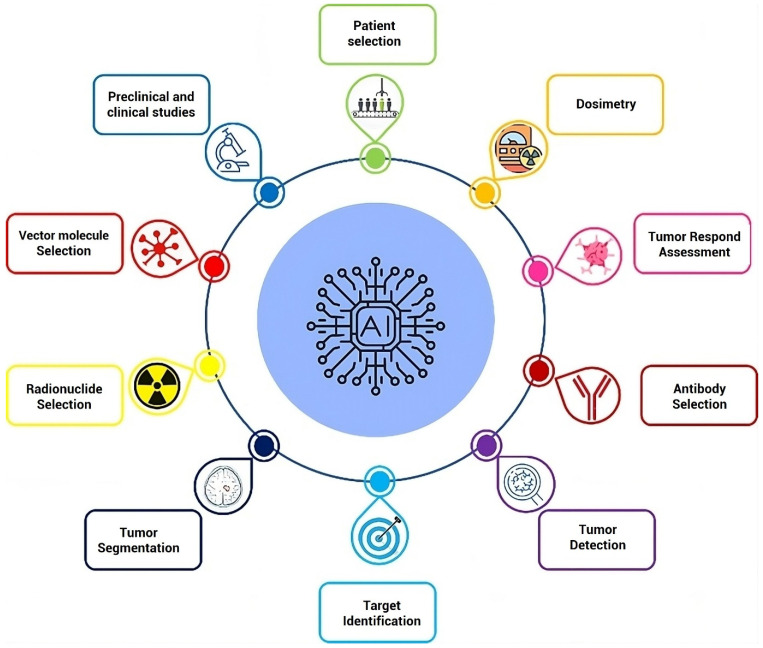
Potential applications of AI in radioimmunotherapy.

The following sections investigate current and future applications of AI in different RIT steps, as shown in Figure 5.

### 10.1. Patient Selection and Treatment Planning

AI in cancer treatment is becoming more widespread and transitioning from specialist research to widely used clinical practice [112]. AI is utilized to plan various cancer treatments (such as radiotherapy) and choose appropriate patients for clinical trials. Manual treatment planning takes a lot of time and is very skill dependent. An important objective of treatment planning standardization is to ensure that all patients receive high-quality care, regardless of the time and expertise of the planner [113]. In a study by Castriconi et al., they use a novel volumetric technique to implement knowledge-based (KB) automatic planning for right- and left-sided entire breast treatment (ViTAT, Virtual Tangential-fields Arc Therapy) imitating traditional tangential fields (TF) irradiation. To train two KB models for patients with left (L)- and right (R)-sided breast cancer, 193 clinical plans delivering TF with wedged or field-in-field beams were chosen in their study. L- and R-side KB models were successfully produced. Out of 30 automatic KB-ViTAT designs, one (3%) and seven (23%) were deemed unsuitable compared with TF for the R and L sides, respectively; following manual start/stop angle refinement, KB-ViTAT designs are well-fitting.

Results imply that for total breast irradiation, fully automatic KB-optimization of ViTAT can effectively replace manually optimized TF planning [114]. In another study by Xu et al., [115] they evaluated the performance of a proton-specific knowledge-based planning (KBP) model in the creation of optimized intensity-modulated proton therapy (IMPT) plans to cure patients with advanced head and neck (HN) cancer. From 73 patients who participated in the study, 53 patients were replanned with optimized IMP, and the remaining 20 patients were used to model validation. Results have shown that all the plans generated were clinically acceptable. The quality of the IMPT programs produced by a broad-scope RPP model (a proton-specific KBP model) is comparable to and, occasionally, even better than the expert plans. The RPP plans showed improved CTV coverage and better sparing for several OARs.

A unique machine-learning model to aid in patient selection for outpatient total shoulder arthroplasty was the focus of Biron and colleagues’ investigation. They employed a random forest machine-learning model to determine which patients had one day or fewer stays. The variables significantly connected with a short or extended stay were then found using multivariable logistic regression. According to findings, 4500 patients were found to have met the requirements for inclusion and have undergone elective total shoulder arthroplasty. The area under the receiver operator curve (AUC) for the machine’s ability to recognize short-stay patients was 0.77. The multivariate logistic regression identified factors linked with a short stay, such as age less than 70 and male sex, and characteristics associated with a more extended stay, such as diabetes and COPD [116].

Recently, Gould et al. [117] developed a machine learning model utilizing non-imaging (EHR) data. This model, which analyzed a dataset of 6505 lung cancer patients and 189,597 control subjects, outperformed the PLCO criteria in predicting lung cancer risk within 9 to 12 months, achieving an AUC of 0.86. Additionally, it enhanced standard eligibility criteria for lung cancer screening, demonstrating that AI-driven analysis of routine clinical data can effectively identify patients suitable for targeted screening initiatives. Employing AI to refine patient selection for screening could prove to be a valuable strategy for facilitating early diagnosis in the future [118].

### 10.2. Dosimetry

In the rapidly evolving field of RPT dosimetry, AI has emerged as a transformative tool, enabling significant advancements in predictive and patient-specific dosimetry. By leveraging AI on pre-therapeutic imaging data, the feasibility of predictive dosimetry has been greatly enhanced, which is crucial for patient stratification and personalized treatment planning in the precision medicine era. For instance, previous studies have shown that machine learning algorithms can accurately predict absorbed doses using baseline PET images prior to administering ^177^Lu-DOTATATE in patients with neuroendocrine tumors, underscoring AI’s potential to revolutionize treatment strategies by making them more tailored to individual patients’ needs [119]. Moreover, recent research has further expanded AI’s role in RPT by demonstrating its effectiveness in image-based predictive dosimetry for ^177^Lu-PSMA therapies in metastatic castration-resistant prostate cancer (mCRPC) patients. These studies have highlighted AI’s capacity to predict voxel-wise dosimetry with a high degree of accuracy, taking into account the intra-organ heterogeneity that is crucial for optimizing therapeutic outcomes in mCRPC treatments [120,121]. These findings collectively illustrate the growing importance of AI in enhancing the precision and efficacy of RPT, paving the way for more personalized and effective treatment regimens.

Recently, Brosch-Lenz et al. [122] reviewed the transformative role of AI in dosimetry for RPT. They discussed how AI has the potential to enhance the accuracy, efficiency, and integration of dosimetry into clinical workflows, which is crucial for personalizing treatment in nuclear medicine. The authors elucidated the complex and time-consuming nature of dosimetry, which involves multiple steps such as quantitative imaging, segmentation of organs and tumors, fitting time-activity curves, and converting these into absorbed doses. They highlight recent advances where AI has been applied to various stages of this workflow, particularly in improving image acquisition speed, segmentation accuracy, and dose calculation processes. The authors conclude that AI holds significant promise in overcoming current limitations, thereby enabling more routine use of patient-specific dosimetry in clinical practice, ultimately contributing to the optimization of RPT for better patient outcomes.

Personalized dosimetry plays an increasing role in nuclear medicine, aligning with the broader movement toward personalized medicine. The integration of AI is driving significant advancements in several areas, such as building theranostic decision trees, ensuring compliance with dose limit, radiation dose monitoring, and dose reduction strategies [123,124]. These advancements are essential for achieving more accurate dose maps, which are critical for tailoring treatments to individual patients in nuclear medicine. Precision in dose estimation is key to minimizing the risk of radiation-induced toxicity, thereby enhancing the safety and efficacy of treatments [125]. In the realm of internal dosimetry, several methodologies are employed to meet the diverse needs of patient care. Monte Carlo simulations, widely regarded as the gold standard, offer unparalleled accuracy in modeling complex radiation interactions within the human body. However, their computational intensity can pose practical challenges, particularly in time-sensitive clinical settings. The Medical Internal Radiation Dose (MIRD) system, which is more commonly used in clinical practice, provides a reliable method for dose calculation based on organ-level assessments. However, the MIRD system’s approach is based on the assumption of a uniform distribution of radioactive activity within a spherical volume, which can lead to inaccuracies when dealing with tumors or tissues that exhibit heterogeneous characteristics. [126]. Voxel-based methods, such as dose point kernel and voxel S-value (VSV) techniques, offer a more refined approach by allowing for the calculation of dose distributions at the voxel level, taking into account the variability in tissue composition and activity distribution. While these methods provide a more detailed and accurate assessment compared to organ-based systems, they are still subject to limitations, particularly in tissues with complex structures or heterogeneous activity distributions. Despite these challenges, the ongoing integration of AI and other advanced computational techniques holds promise for further improving the precision and applicability of personalized dosimetry in nuclear medicine [127,128,129].

As another example of AI-based RPT dosimetry, Jackson et al. [130] used a 3D CNN architecture to automate the monitoring of absorbed doses in the kidneys of patients undergoing ^177^Lu-PSMA therapy. They used post-treatment SPECT imaging to provide organ-level dosimetry to estimate renal radiation doses from radiopharmaceutical therapy. Akhavanallaf et al. [131] used ResNet architecture for precision dosimetry in nuclear medicine procedures. In this research, the idea behind the voxel-scaled MIRD approach has been extended by the prediction of specific S-values according to the map of density derived from CT images and then calculating the cumulated activity map from the predicted particular kernels. For the prediction of the energy deposited in the volume surrounding a unit radioactive source in the center of the kernel, a physics-informed deep neural network (PIDNN) model was designed. For the input channel, they used a density map, and for the output channel, Monte Carlo-based deposited energy maps of the given radiotracer were used, which were called specific S-value kernels. Götz et al. [132] and Lee et al. [133] present a study on “Deep-dose,” a novel method using a deep convolutional neural network (CNN) for voxel-based dose estimation in personalized internal dosimetry. The method leverages PET and CT image patches as inputs, with ground truth data from Monte Carlo simulations, to generate dose rate maps. The CNN-based approach demonstrated significant improvements in accuracy and speed compared to conventional methods like VSV kernel convolution and organ-based dosimetry, particularly in areas with heterogeneous tissue densities such as the lungs. This approach showed a whole-body voxel dose error of 2.54 ± 2.09%, markedly better than other methods, and reduced computation time significantly.

AI holds significant potential in advancing dosimetry for radioimmunotherapy (RIT), both in predictive and post-treatment contexts. For predictive dosimetry, AI algorithms can be harnessed to analyze a wide array of data, including clinical records, imaging results, pharmacokinetic profiles, physiological parameters, antibody status, and immunological data, to accurately forecast the dose required for effective treatment [134]. These predictions can be refined by incorporating data collected from human subjects, animal models, in vitro studies, and even organ-on-a-chip platforms, ensuring that AI models are trained on diverse and representative datasets. However, further studies with sufficiently large sample sizes are essential to validate these AI-driven predictive models and ensure their reliability and applicability in clinical settings [135].

In post-treatment dosimetry, AI can revolutionize the workflow by automating and enhancing various stages of the process. AI-driven tools can be employed for image segmentation, enabling precise delineation of tumors and organs at risk. These tools can also assist in image processing and registration, ensuring that multi-modal images are accurately aligned for comprehensive dose assessment [136]. AI is also critical in motion estimation and correction, allowing for adjustments in dose calculations based on patient movement during imaging or treatment, thereby improving the accuracy of the dosimetry. Additionally, AI can enhance scatter and attenuation estimation and correction, which are essential for obtaining accurate dose distributions from imaging data. Furthermore, AI can augment data through techniques such as data synthesis and augmentation, thereby enriching the dataset available for analysis and improving the robustness of dosimetric calculations [137]. In the fitting of pharmacokinetic data and estimation of dosimetric parameters, AI algorithms can provide more accurate and faster results than traditional methods. Finally, AI can facilitate detailed dose analysis and reporting, offering clinicians comprehensive insights into the delivered dose distribution, which is crucial for assessing treatment efficacy and planning future interventions. As AI continues to evolve, its integration into radioimmunotherapy dosimetry promises to enhance precision, efficiency, and patient outcomes [138].

Mansouri et al. [139] developed a hybrid deep learning model based on a transformer architecture for personalized voxel-level dosimetry in 177Lu-DOTATATE radiopharmaceutical therapy. The model integrates a multiple voxel S-value (MSV) approach with deep learning (DL) to enhance dosimetric accuracy, closely approximating Monte Carlo (MC) simulations, the gold standard in dosimetry. Using data from 22 patients across 50 therapy sessions, the DL model demonstrated superior performance compared to traditional MIRD-based methods, particularly in heterogeneous tissue regions. The hybrid model not only reduced computational time significantly but also achieved lower voxel-wise errors and high gamma pass rates, making it a promising tool for clinical implementation in personalized dosimetry.

### 10.3. Tumor Detection, Segmentation, and Classification

Tumor detection and segmentation using AI can be critical in RIT. AI significantly impacts image processing through diverse methodologies like machine learning, deep learning, and neural networks, revolutionizing the landscape of medical imaging practices from image acquisition to diagnostic procedures. The evolution of multiple machine learning and deep learning algorithms aims to support doctors by automating a range of tasks, including lesion detection and the quantification of medical images [140]. The manual identification, categorization, and delineation of tumors are resource-intensive and time-consuming. Consequently, automated methodologies are greatly valued for their efficiency and effectiveness in this domain [141]. For example, identification of brain tumors is conducted using a range of techniques involving transfer learning and deep learning approaches, notably utilizing CNN architecture for segmenting and categorizing [142]. Within the field of radiology, these methodologies have the potential to significantly contribute to more efficient and reliable diagnosis, staging, treatment response monitoring, and overall patient management by clinicians [142].

Narayana and Reddy proposed a segmentation method using a genetic algorithm (GA) based on median filtering. They utilized features derived from the gray-level co-occurrence matrix (GLCM) alongside a support vector machine (SVM) classifier, which was evaluated using the Harvard medical image dataset and achieved an accuracy of 91.23%. Similarly, Minz and Mahobiya employed median filtering for noise reduction and threshold segmentation to improve tumor detection efficiency on a publicly available brain tumor MRI dataset, with their method attaining an accuracy of only 89.90%. Liu et al. explored a deep learning (DL) model aimed at diagnosing brain diseases, utilizing a landmark-based approach for data-driven learning. The study utilized three datasets: ADNI-1, ADNI-2, and MIRIAD. This landmark-based DL technique enables the model to learn from images comprehensively by extracting both local and global features. On the ADNI-1 dataset, it recorded an impressive accuracy of 92.75% and an AUC of 97.16%. For the ADNI-2 dataset, the model achieved an accuracy of 91.09% with a 95.86% AUC. Similarly, on the MIRIAD dataset, it also reached an accuracy of 92.75% and a 97.16% AUC [143].

Meanwhile, there is a need to observe best practice guidelines for AI algorithm development [144] and validation [145] and a need to develop trustworthy AI ecosystems [146] for routine use of AI in clinical practice. Numerous algorithms have been developed expressly to distinguish between areas linked to lung nodules. Thresholding, the region growth algorithm, morphological filters, connected component analysis, and the boundary tracking algorithm are the primary traditional methods. The effectiveness of lung segmentation has been further enhanced by several better ways based on conventional methods, which have also optimized the weaknesses of the classic methods [147]. Two-dimensional (2D) region-growing algorithms were the main focus of Shi et al. [148]. The smoothed slice was converted into a binary image by applying an optimal threshold using a method based on seed-based random walks, which made it possible to separate the thorax region from the lung region.

HLA plays a crucial role in tumor detection by the immune system. Bulik et al. [149] created a comprehensive dataset that includes HLA types and HLA peptides from various cancer tissues, which has been published and can be utilized to train the complete mass spectrometry deep learning model EDGE, previously validated in patients with non-small-cell lung cancer (NSCLC). Recently, two innovative deep learning approaches, MARIA and MixMHC2pred, have emerged, significantly enhancing the accuracy of MHC-II predictions. MARIA is developed using not only in vitro binding affinity data but also naturally presented MHC-II ligands identified through liquid chromatography-tandem mass spectrometry (LC-MS/MS) and gene expression levels. It employs a recurrent neural network (RNN) to generate a presentation score [150].

Ranjbarzadeh et al. conducted a study in which they built a cascade convolutional neural network (C-ConvNet/C-CNN) for brain tumor segmentation. The network benefits from the characterization of four MRI modalities: T1, T1c, T2, and FLAIR. First, they suggested a preprocessing method to operate on a smaller portion of the image rather than the entire image, which reduces computing time and addresses overfitting issues in a cascade deep learning model. The segmentation was then carried out in two parts. In the second stage, an effective and straightforward C-ConvNet/C-CNN is suggested, which deals with a smaller portion of brain pictures in each slice. This C-CNN model uses two distinct methods to mine local and global characteristics. In the entire tumor segmentation, the mean sensitivity and mean dice score were, respectively, 0.9386 and 0.9203 [151].

### 10.4. Drug Design and Discovery

The overwhelming amount of data and the requirement for quick analysis present enormous problems to biology, chemistry, and medicine. Given data noise, non-linearity, and temporal dynamics, computational intelligence (CI) techniques, including artificial neural networks, fuzzy systems, and evolutionary computation, are being employed more frequently to address this issue. These techniques can be used alone or in conjunction with conventional statistical methods to create reliable models of processes [152]. Complex biological systems can provide valuable information during development and illness. With the help of numerous “omics” and smart technologies, this information is now being systematically quantified and mined at a level that has never been seen before. The pharmaceutical industry, whose goal is to find credible therapeutic hypotheses from which to produce medications, faces both obstacles and opportunities due to the emergence of these high throughput methods to biology and illness.

In general, computational modeling and simulation approaches focus on the structure and behavior of compounds. Binding affinity of a radiopharmaceutical for its target is predicted by structural models, while the focus of behavioral approaches is on pharmacokinetics. Both structural and behavioral approaches are indispensable and mutually supportive. This is because an effective radiopharmaceutical, which can be applied in diagnostics or therapeutics, must exhibit precise specificity and strong binding affinity for its target. Additionally, it should demonstrate optimal biodistribution, stability, practical effective half-life, and a desirable clearance profile [153]. ML-based software and algorithms are being developed and used at all stages of drug design and development, including finding novel targets, increasing understanding of disease mechanisms, increasing understanding of disease and non-disease phenotypes, improving small-molecule compound design and optimization, providing more substantial evidence for target–disease associations, developing new biomarkers for prognosis, progression, and drug efficacy, improving analysis of biometric and other data from patient monitoring and wearable devices, enhancing digital pathology imaging, etc. [154].

### 10.5. Target Identification and Validation

Being unsafe and ineffective are two main reasons for the failure of new drugs to be used in the clinic. A crucial initial step in creating an effective radiopharmaceutical is choosing a target. A target refers to various biological entities, such as proteins, genes, and RNA. The optimal target is effective, safe, matches clinical and commercial goals, and selectively expressed on disease tissues in high copy numbers and should be accessible for radiopharmaceutical binding. After identifying a target, it needs to be validated, and this can be done by using physiologically related ex vivo and in vivo models. Although clinical trials will ultimately validate the target, early target validation is essential to concentrate resources on initiatives with a good chance of success. A wide range of techniques, including database searching, proteomics, and transcriptomics, may be utilized in the era of “omics” technology to validate existing targets and discover new ones.

Making ligands with a high affinity and high specificity for the target is the next stage in developing radiopharmaceuticals once the target has been determined [154,155,156]. Studies suggest that machine learning (ML) approaches in biological analysis can effectively manage large volumes of diverse and intricate molecular data while uncovering features or relationships within biological networks. Therefore, it is essential to create additional ML-based algorithms for biological analysis that facilitate precise target identification and drug discovery in cancer research. Cantini et al. created a workflow for network-based biological analysis that consolidates various layers of genomic data, such as transcription factor co-targeting, miRNA co-targeting, protein–protein interactions, and gene co-expression, into a comprehensive biological network. Subsequently, they utilized a consensus clustering algorithm—an ML-based approach that segments the network into functional sub-modules—to analyze the identified network communities and uncover potential cancer driver genes. Their findings indicated that F11R, HDGF, PRCC, ATF3, BTG2, and CD46 could function as oncogenes and serve as promising biomarkers for pancreatic cancer [157].

### 10.6. Radionuclide Selection Process

In the development of radiopharmaceuticals, the choice of radionuclide is critical and depends largely on whether the application is diagnostic or therapeutic. Despite the limited number of radionuclides that meet the criteria for nuclear medicine, selecting the most appropriate one for a specific application can be complex [158]. This decision requires careful consideration of several parameters, including the radionuclide’s physical and chemical characteristics, availability, production simplicity, cost, and the feasibility of purification and isolation. When designing radiopharmaceuticals, it is necessary to evaluate the radionuclide’s decay mode—whether it emits alpha (α), beta (β), or gamma (γ) radiation—as this influences its suitability for either therapeutic or diagnostic use. Other important factors include the radionuclide’s specific activity, its biodistribution and uptake in biological tissues, the energy of the emitted radiation, and the clearance rate of the radiotracer from both target and non-target tissues [159].

The efficiency of drug delivery to the target site is also a crucial factor that impacts the overall effectiveness of the radiopharmaceutical. Additionally, it is important to align the radionuclide’s half-life with the pharmacokinetics of the targeting agent. The half-life should be sufficiently short to minimize patient radiation exposure while being long enough to allow for the necessary diagnostic or therapeutic procedures. Compatibility between the radionuclide and the vector molecule’s chemistry is essential to ensure stable labeling without compromising biological function. The radionuclide labeling process should achieve high yield, provide sufficient specific activity, and ensure stability both in vivo and in circulation to maintain the radiopharmaceutical’s safety and efficacy [160].

### 10.7. Designing and Selection of the Targeting Molecule

Small organic molecules, peptides, proteins, antibodies, antibody fragments, carbohydrates, lipids, nucleic acids, microspheres, and, more recently, organic and inorganic nanoparticles having pertinent biological characteristics are examples of radiopharmaceutical vector molecules or delivery systems. Different types of vectors have their advantages and disadvantages. The desired diagnostic or therapeutic function, delivery efficiency, in vivo metabolism, and pharmacokinetics affect the choice of the delivery system [161].

To design radiopharmaceuticals incorporating different kinds of vector molecules, the CADD method can be considered a helpful tool. Pharmacological activities and chemical properties can be predicted using computational methods, and potentially valuable molecules for different targets can be detected using in silico methods. To accelerate the design and optimization of lead compounds in developing drug and vector molecules in radiopharmaceutical design, various computational methods including ML models, 3D-QSAR approaches, molecular docking, chemical similarity searching, and pharmacophore modeling can be helpful [162].

### 10.8. Radiolabeling

The radionuclide used ultimately determines the labeling strategy utilized to obtain a radiopharmaceutical. Some radionuclides require indirect radiolabeling of the vector molecule, while others allow direct radiolabeling. The most used approach for radiohalogens is direct radiolabeling, which attaches the radionuclide either covalently or ionically to the rest of the molecule (the radio-halogenation method). In the indirect labeling technique, chelating agents combine metallic radionuclides to produce stable complexes under physiological circumstances. The in vivo complex stability and biodistribution of radiopharmaceuticals are affected by the charge and hydrophilicity of the chelator. For the radiopharmaceutical to have the best possible selective target uptake, the chelator must be matched to the selected metallic radionuclide and the biological targeting molecule [163]. Radiolabeling procedures maximize labeling yield, maintain target specificity, produce high radiochemical purity, transport, and use, and assure radiopharmaceutical stability during storage [160].

To choose a good chelator and prevent mistakes, it is essential to comprehend the coordination chemistry of the selected radionuclide. Metal complexes’ structural characteristics, energies, and electronic properties have all been calculated with varied degrees of accuracy using molecular mechanics (MM), quantum mechanics (QM), and combined MM/QM techniques [164,165]. The physicochemical characteristics of metal ion complexes, such as stability constants, binding affinities, lipophilicities, and solubilities, can be precisely predicted by data-driven ML techniques, such as quantitative structure-activity relationships (QSAR) and quantitative structure-property relationships (QSPR) [166,167]. In addition to identifying the most suitable chelator, determining the ideal target molecule and pinpointing the optimal labeling site based on metabolic stability and radiochemical accessibility are crucial aspects of radiochemistry. ML offers extra resources for retrosynthetic analysis and holds promise as a core tool for building a retro-radiosynthesis tool. Just like conventional retrosynthetic analysis tools, template reactions for radiolabeling can be established. However, the differentiating factor between traditional retrosynthetic tools and any potential tool for retro-radio-synthesis lies in the scoring function defined for radiochemistry. Any program designed for this purpose will aim to maximize feasibility, activity, and specific activity while minimizing the time required for the crucial radiolabeling step and metabolic breakdown [168].

### 10.9. Pre-Clinical Trials

New drugs must be studied to determine their effects on non-target tissues and drug performance. The mechanism of action of radiopharmaceuticals is usually radiation-induced cell damage and has no pharmacological activity on the target tissues [169]. Several properties like suitable kinetics, different uptake by changes in target expression, selectivity, effectiveness, low toxicity to healthy tissues and organs, and cell-killing activity in the case of therapeutic radiopharmaceuticals should be determined in preclinical studies [170,171]. ADMET (absorption, distribution, metabolism, excretion, and toxicity), efficacy, and pharmacokinetic properties can be investigated in preclinical drug design studies, which can be in silico, in vivo, and in vitro [172]. Structure-based, system-based, and ligand-based methods are three in silico methods used for designing drugs [173,174]. In the early stages of preclinical radiotracer research, high throughput approaches are used to forecast pharmacokinetic characteristics and give data for training ML models. Choosing an appropriate computational-aided drug design (CADD) method depends on the availability of structural information for the target protein. For structure-based drug design (SBDD), experimental techniques like nuclear magnetic resonance or X-ray crystallography are typically used to obtain the protein’s structural data. If this information is not accessible, in silico methods, such as homology modeling or ab initio modeling, can be employed to estimate the protein’s 3D structure. Once the structure is determined, virtual screening and molecular docking based on that structure can be performed. If the structure remains undetermined and high-quality predictions using in silico techniques are not feasible, the ligand-based drug design (LBDD) approach can be used as an alternative. This strategy requires knowledge of previously identified active compounds for the target protein. Fortunately, many compounds, documented for treating various diseases, are available in public databases, unless the target protein is entirely new [175]. Velázquez-Libera et al. [176] outlined a hybrid approach that combines structural and ligand-based methods to examine the structural factors influencing the affinity of various compounds for the human sigma-1 receptor (S1R), a significant target for treating neuropsychological disorders. They identified an effective S1R agonist, RC-33, as a promising neuroprotective agent. The authors conducted a computational analysis of how RC-33 and its new derivatives interact with the S1R active site. They employed various in silico techniques, including docking, interaction fingerprints, and receptor-guided alignment for three-dimensional quantitative structure-activity relationship (3D-QSAR) studies, to explore potential mechanisms of action of these compounds. The findings presented could aid in the design of new S1R modulators. Baillif et al. [177] conducted a computational study utilizing a public dataset of compound-induced transcriptomic data to forecast the potential activity of compounds against 69 drug targets. The researchers evaluated the effectiveness of machine learning models developed with transcriptomic data alongside computational tools generated from Morgan fingerprints. Interestingly, active compounds directed at a particular target exhibited similar signatures across one or more cell lines, irrespective of the chemical structure similarities among the chosen active chemical entities. For 25% of the evaluated tasks, the random forest models using transcriptomic signatures demonstrated comparable or superior performance compared to those based on Morgan fingerprints. The data derived from compound-induced transcriptomics presents a promising opportunity for predicting targets based on similarities in cellular responses, helping to bypass the limitations of chemical space found in quantitative structure-activity relationship (QSAR) models.

### 10.10. Clinical Trials

**Figure 6:**
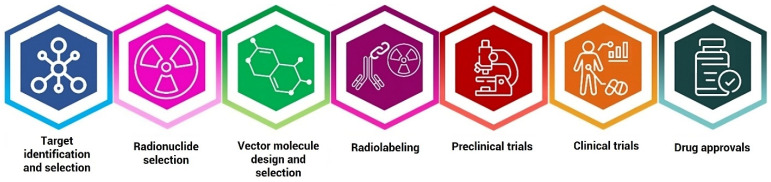
The process of designing radiopharmaceuticals.

AI is not a replacement for traditional radiopharmaceutical design approaches, but a supplement to them. As mentioned before, the efficacy of radiopharmaceuticals developed in silico must be verified by in vivo research and clinical trials. In silico techniques can significantly shorten the time frame and lower the cost of radiopharmaceutical development to make radiopharmaceuticals more available for non-invasive imaging and therapy in various targets. The development in structural chemistry can be used to speed up the design of a broad range of radiopharmaceuticals using AI for clinical use. AI techniques have proved very useful for designing CNS radiotracers, whereas BBB makes it more difficult for radiopharmaceuticals to reach their target. There is currently a lack of a well-established systematic strategy for integrating in silico approaches into radiopharmaceutical design [153]. Patient enrollment accounts for one-third of the timeline in clinical trials. The success of these trials largely depends on recruiting appropriate patients, as unsuitable recruitment is linked to 86% of failure cases. AI can aid in identifying a targeted patient population for recruitment in phase II and III trials by analyzing individual genome-exposome profiles, which can facilitate the early identification of potential drug targets in the selected patients [178]. By examining electronic medical records, AI has the potential to anticipate the likelihood of participants dropping out of clinical trials. Instead of excluding those who might drop out, initiatives in the cardiovascular therapeutic area have aimed to specifically engage these individuals and provide them with additional education to support sustained participation. These strategies can help reduce overall sample sizes, thereby requiring fewer participants for the trial. in addition, research has shown that ML prediction models could decrease cancer mortality rates by 15–25% across various clinical trials. Such ML algorithms, which facilitate clinical outcome predictions based on environmental and genetic factors, can be developed from extensive biological databases that link drug-related predictive biomarkers from interventional studies with data on progression-free and overall survival, categorized by the molecular profiles of tumors. Moreover, a study focused on non-small cell lung cancer trials evaluated the effectiveness of biomarker status, along with other complex factors, in predicting tumor response and survival rates using ML-based tumor growth models [179]. Figure 6 illustrates all of these steps simultaneously.

### 10.11. Tumor Response Assessment

In addition to patient selection and treatment planning, AI is also used to evaluate treatment responses, assisting oncologists in providing better treatment advice. Such models would need thorough, ideally prospective clinical validation, which is still pending for a histopathology-specific predictive AI-based biomarker [180]. Madabhushi et al. developed a machine learning-based model to predict response to immunotherapy using computer-extracted features of cancer nuclei from hematoxylin and eosin (H&E) stained images of non-small cell lung cancer [181]. Moreover, Farahmand et al. [182] introduced a unique convolutional neural network (CNN) method for HER2 status and trastuzumab treatment response prediction in HER2+ breast cancer. A total of 188 H&E full slide pictures that the pathology team had manually tagged for tumor regions of interest were used to train the CNN classifier. In cross-validation of the HER2 status at the slide level, the classifier obtained an area under the curve of 0.90. Pre-treatment samples from 187 HER2+ patients who underwent trastuzumab therapy were also employed to train classifiers. The AUC of the classifier was 0.80 in fivefold cross-validation. Predicting well-established indicators of treatment response, like microsatellite instability (MSI), is a further step [180]. Yamashita et al. examine the possibilities of a deep learning-based system for automatic MSI prediction directly from whole-slide images (WSIs) stained with hematoxylin and eosin (H&E). Utilizing 100 H&E-stained WSIs (50 with microsatellite stability [MSS] and 50 with MSI), a deep learning model (MSINet) was created. The model was externally verified using 484 H&E-stained WSIs (402 cases with MSS and 77 with MSI; 479 people) from The Cancer Genome Atlas and internally validated using a holdout test set (15 H&E-stained WSIs from 15 patients; seven cases with MSS and eight with MSI). On the holdout test set from the internal dataset, the model’s AUC was 0.931 (95% CI 0771-1000), and on the exterior dataset, it was 0.779 (0720-0838). Using a sensitivity-weighted operating point on the external dataset, the model produced results with an NPV of 93.7% (95% CI 90.3–96.2), a sensitivity of 76.0% (64.8–85.1), and a specificity of 66.6% (61.8–71.2) [183]. Peng et al. [184] used a long short-term memory (LSTM) recurrent neural network (RNN) model to clarify the potential benefits of combining PULSAR and PD-L1 blockade immunotherapy using experimental data from a syngeneic murine cancer model based on Lewis lung carcinoma (LLC). They simulated the treatment response by treating irradiation and anti-PD-L1 as sequential external stimuli. Their results indicate that the model can effectively simulate tumor growth by incorporating various factors such as timing and dosage, and it offers mechanistic insights into the “causal relationship” of the combined treatment, presenting a completely new perspective. They suggest that this model can be employed for in silico analysis, enabling the exploration of novel treatment combinations to enhance therapeutic results.

## 11. Navigating the Challenges of AI Integration in Radioimmunotherapy: Technological, Ethical, and Economic Considerations

The drawbacks of AI in radioimmunotherapy underscore the enormous technological, ethical, clinical, economic, and regulatory problems connected with incorporating these cutting-edge innovations into healthcare settings. While AI has the potential to improve diagnostic accuracy and treatment efficacy, its use raises issues about the validity of outcomes, possible unconscious biases in decision-making, and the consequences for patient safety and autonomy. These challenges are more obvious in radioimmunotherapy, where accurate dose and patient-particular considerations are critical for effective results. One important issue is the reliance on high-quality, diversified datasets to train AI algorithms. When these models are built with inadequate demographic data, they might perform badly when applied to larger populations, potentially worsening existing health inequities [185]. In addition, there is an issue of over-reliance on AI, which could reduce healthcare professionals’ conventional diagnostic abilities, upsetting the balance between human expertise and machine help [186,187]. The absence of uniform testing and regulatory structures for AI technologies further complicates the situation, raising questions about their dependability and reliability in clinical practice [188]. Ethical considerations loom big, particularly in terms of informed approval and patient autonomy. Many patients may not fully comprehend the importance of AI in their therapy, and the complexity of AI algorithms can impede direct interaction between healthcare providers and patients [189]. Furthermore, issues of privacy of information and algorithmic fairness are crucial, as biased AI models might perpetuate healthcare inequities, disproportionately impacting underrepresented populations [190]. Finally, the budgetary constraints to deploying AI technologies in radioimmunotherapy can result in inequities in access, especially for smaller healthcare facilities. The initial and ongoing expenses of AI systems can be exorbitant, raising worries about equitable access to sophisticated therapies and increasing existing disparities in healthcare delivery [191]. Addressing these drawbacks is critical to ensuring that the incorporation of AI into radioimmunotherapy is both profitable and moral, resulting in equal healthcare results for all patients.

## 12. Modeling Radioimmunotherapy: Is It Too Early?

It is still difficult to model the entire RIT process, as it consists of several parts, and despite the prior efforts [192,193,194,195,196,197], there are still many unanswered questions in this field. One of the problems in modeling RIT is the paucity of clinical data related to this treatment method. RIT is an expensive method that cannot be applied to all cancer patients. Although the solution to this problem has not yet been identified, an extension of physics-informed neural networks called biologically informed neural networks (BINNs) was presented and utilized to extract the fundamental dynamics of biological systems from sparse experimental data [198]. Building mechanistic predictive models using biologically directed neural networks is a novel way to combine machine learning and cancer biology. This platform for biological discovery may be helpful in various cancer prediction and discovery activities [199].

Recently, Shaier et al. introduced a type of neural network called disease-informed neural network (DINN) that can efficiently anticipate the spread of infectious diseases. The algorithms may be used for complex models (such as those with spatial dependencies). According to their findings, DINN is a solid and trustworthy contender that may be utilized as an inverse method to define and discover the parameters of compartmental models. Since RIT is a compartmentalized process, a disease-informed neural network can potentially be effective in modeling this process. Additionally, even though the DINNs provided are demonstrated to be trustworthy and robust, they can be slow to train in specific situations, and there is no known theoretical guarantee of commensurate error bounds [200].

## 13. Conclusions

Our exploration underscores the potential of radioimmunotherapy (RIT) and the pivotal role of artificial intelligence (AI) in reshaping cancer treatment paradigms. Traditional cancer therapies have faced limitations, prompting the emergence of RIT as a powerful, targeted alternative, albeit with current cost constraints hindering widespread implementation. AI is emerging as a transformative tool capable of customizing cancer treatment strategies by analyzing factors such as cancer type, stage, and patient demographics to recommend the optimal combination of antibodies, radio-conjugates, and dosages for individual patients. Integration of AI across various facets of RIT offers new possibilities for advancing personalized cancer care.

Despite existing challenges, including complexities of RIT computational modeling, ongoing research endeavors and technological advancements are propelling us toward a future where AI-driven RIT models can guide treatment decisions with precision and efficacy. Given the contributions of pioneers, efforts, and collaborations, AI has the opportunity to help empower us to navigate the complexities of cancer treatments with greater clarity and effectiveness.

Additionally, navigating the challenges of AI integration in radioimmunotherapy is critical. We must address the technological, ethical, clinical, economic, and regulatory issues associated with AI use to ensure that it enhances rather than undermines patient outcomes. By tackling these challenges, we can work towards a more equitable healthcare landscape that benefits all patients in the realm of radioimmunotherapy.

## Figures and Tables

**Table 1:** A listing of therapeutic radionuclides used in radioimmunotherapies.

	LET in Water (keV/µm)	Energy (MeVmax)	Range	Half-Life	Clinical Experience Example
Beta emitter					
Yttrium-90	2.00	2.28	11.3 mm	2.7 days	[35]
Lutetium-177	0.28	0.50	1.8 mm	6.7 days	[36]
Alpha emitter					
Actinium-225	102	6.8	82.2	10 days	[37]
Bismuth-213	102	8.3	60–85 μm	0.8 h	[38]
^212^Pb	99.4	8.8	88.5 μm	10.6 h	[39]
Astatine-211	NA	6.8	NA	7.2 h	[40]
Auger electrons					
Indium-111	NA	19 keV	NA	2.8 days	[41]
Iodine-125	NA	NA	2–500 nm	60.5 days	[42]

## Data Availability

No new data were created or analyzed in this study.

## Abbreviations (Sorted Alphabetically)

ADCC	Antibody-dependent cellular cytotoxicity
ADCP	Antibody-dependent phagocytosis
AI	Artificial intelligence
AML	Acute myeloid leukemia
Bsab	Bispecific antibody
CA	Clearing agent
CDC	Complement-dependent cytotoxicity
DL	Deep learning
DNL	Dock and lock
DTPA	Diethylenetriaminepentaacetic acid
DVH	Dose-volume histogram
EBRT	External beam radiation therapy
HSG	Histidine-succinyl-glycine
IV	Intravenous
LET	Linear energy transfer
LR	Logistic regression
mAb	Monoclonal antibody
MAC	Membrane attack complex
MC	Monte Carlo
MTC	Medullary thyroid cancer
PRID	Pretargeted radioimmunodiagnosis
PRIT	Pretargeted radioimmunotherapy
RECIST	Response Evaluation Criteria in Solid Tumors
RF	Random forest
RIT	Radioimmunotherapy
RPT	Radiopharmaceutical therapy
SVM	Support vector machine
TI	Therapeutic index
